# Plasma levels of adiponectin and chemerin are associated with early stage of atherosclerosis in older adults with type 2 diabetes mellitus

**DOI:** 10.1002/agm2.12087

**Published:** 2019-10-30

**Authors:** Huahua Li, Junkun Zhan, Bin Liao, Yanjiao Wang, Youshuo Liu

**Affiliations:** ^1^ Department of Geriatrics The Hunan Provincial People's Hospital First Affiliated Hospital of Hunan Normal University Changsha China; ^2^ Department of Geriatrics The Second Xiang‐Ya Hospital Institute of Aging and Geriatric Research Central South University Changsha China

**Keywords:** adiponectin, brachia‐ankle pulse wave velocity, chemerin, type 2 diabetes mellitus, vascular stiffness

## Abstract

**Objective:**

Adipokines, such as adiponectin and chemerin, regulate fat metabolism and are critical for the development of atherosclerosis. Investigating the correlations of adiponectin and chemerin with atherosclerosis in older adults with type 2 diabetes mellitus (T2DM) will shed light on the search for new markers for early diagnosis of diabetic atherosclerosis.

**Methods:**

A total of 120 older T2DM patients and nine healthy controls were enrolled in this study. The clinical parameters, such as brachial‐ankle pulse wave velocity (ba‐PWV), adiponectin, and chemerin, were examined and recorded. T2DM subjects were divided into three groups according to ba‐PWV level (high, medium, or low). The data were processed and analyzed by identical methods.

**Results:**

Significantly higher chemerin and lower adiponectin levels were detected in the plasma of T2DM patients than in healthy controls. The plasma levels of chemerin and adiponectin were negatively correlated in T2DM patients. Moreover, chemerin and adiponectin were significantly correlated with body mass index, ankle‐brachial index, insulin, and ba‐PWV. Multiple linear regression analysis revealed that chemerin and adiponectin were independently affecting ba‐PWV.

**Conclusion:**

Adiponectin and chemerin are potential markers for diagnosis and monitoring of early stage of atherosclerosis in older adults with T2DM. Further clinical investigations are required to confirm these markers.

## 1 INTRODUCTION

Atherosclerosis, as one of the most serious complication of diabetes, is often found at the early phase of diabetes when vascular endothelial cell injury takes place.^1^ Compared with nondiabetic subjects, type 2 diabetes mellitus (T2DM) could favor a high risk of macrovascular disease.^2^ Adipokines are cytokines (cell‐signaling proteins) secreted by adipose tissue, including adiponectin, leptin, interleukin‐6, and chemerin.^3^ Several studies have indicated that adipokine imbalance can lead to atherosclerosis and this is critical in the development of diabetes.^4^, ^5^ Obese adipose tissue secretes high amounts of chemerin and low levels of adiponectin, which leads to a pathogenic and pro‐inflammatory environment for the development of atherosclerosis and diabetes.^3^, ^4^ Previous studies have suggested that serum adipokine levels may be helpful for the early diagnosis of atherosclerosis.^1^, ^6^ Pulse wave velocity (PWV), including carotid‐femoral PWV and brachial‐ankle PWV (ba‐PWV), is the gold standard to assess arterial stiffness, as well as in identification of early stage of atherosclerosis and cardiovascular events, but its clinical application is limited due to the operation's complexity.^7^, ^8^ Still, whether the plasma levels of chemerin and adiponectin could reflect the degree of atherosclerosis in T2DM is still unknown.^9^, ^10^, ^11^ In this study, we divided all patients with T2DM into three groups according to their ba‐PWV values. The levels of adiponectin and chemerin together with other clinical parameters were measured and compared among all groups. Overall, our findings indicated that the plasma levels of adiponectin and chemerin correlated with the development of vascular stiffness, which may lead to atherosclerosis in T2DM.

## 2 METHODS AND SUBJECTS

### 2.1 Patients and samples

This cross‐sectional study included 129 subjects (aged over 65 years), including nine healthy older adults and 120 older patients with T2DM who had undergone medical treatment from June 1, 2015 to December 1, 2015 in the general department of the Mawangdui Campus of Hunan Province People's Hospital. Diabetes had been diagnosed according to the 1999 World Health Organization Diabetes Diagnosis Standard. We excluded patients with acute or chronic infectious disease, cerebrovascular disease, malignant tumor, severe liver and/or kidney disease, acute myocardial infarction, immune system diseases, surgery history or trauma within the previous half‐year, and administration of rosiglitazone medicine. The present study was approved by the Research Ethics Committee of Hunan Normal University. Before enrollment, written informed consent was acquired from each individual. Some basic clinical characteristics of all T2DM patients are shown in Table 1.

**Table 1:** Clinical parameters of all T2DM patients and healthy controls

Parameter	N = 120, Male/Female = 70/50				Healthy controls
	Minimum	Maximum	Mean	SD	
Age (y)	65.00	97.00	83.83	6.50	—
History of T2DM (y)	10.00	32.00	21.40	4.94	—
SBP (mm Hg)	106.00	170.00	140.41	13.11	<140
DBP (mm Hg)	55.00	98.00	76.67	9.19	<90
BMI (kg/m^2^)	18.49	34.55	23.97	3.12	18.5‐24.99
Waist circumference (cm)	75.00	115.00	97.12	8.07	—
Hip circumference (cm)	77.00	120.00	100.71	8.65	—
Waist‐to‐hip ratio	0.81	1.14	0.97	0.07	Male < 0.9; Female < 0.8
GPT (U/L)	5.00	51.00	20.48	8.39	0‐40
AST (U/L)	10.00	44.00	23.02	7.56	0‐40
BUN (mmol/L)	2.50	25.00	6.23	2.41	1.7‐8.3
Serum creatinine (µmol/L)	37.00	229.00	85.44	25.43	35‐105
Trioxypurine (µmol/L)	204.00	455.00	310.28	57.62	150‐360
GH (%)	3.70	11.50	6.03	1.27	3.6%‐6.0%
CRP (mg/L)	0.20	21.30	3.00	2.80	0‐6
Albumin (g/L)	31.00	49.00	38.19	3.47	35‐55
BNP (pg/mL)	103.00	3100.00	1033.54	795.13	<125
LDL‐C (mmol/L)	0.80	6.10	2.28	0.96	0‐3.36
HDL‐C (mmol/L)	0.45	1.89	1.10	0.30	0.78‐1.81
Triglyceride (mmol/L)	0.32	3.20	1.43	0.58	0.45‐1.69
Total cholesterol (mmol/L)	1.98	7.70	3.73	0.95	3.4‐5.2
BMD *T* value	−6.60	−2.20	−1.56	1.53	≥−1.0
ABI	0.60	4.40	1.01	0.42	0.9‐1.3
C peptide (ng/mL)	0.18	4.82	1.92	1.02	0.78‐5.19
Insulin (pmol/L)	0.45	788.00	172.60*	170.86	12.9‐84.7
Chemerin (ng/mL)	90.60	196.92	136.39	20.98	—
Adiponectin (µg/mL)	8.16	44.41	12.75	4.56	3‐30
FBG (mmol/L)	3.90	14.40	6.24	1.94	3.89‐6.11
IRI	0.02	59.92	7.84*	10.10	<1.0
ba‐PWV (cm/s)	1450.00	4292.00	2391.78*	509.00	<1400

Abbreviations: ABI, ankle‐brachial index; AST, aspartate aminotransferase; ba‐PWV, brachial‐ankle pulse wave velocity; BMD *T* value, bone mineral density *T* value; BMI, body mass index; BNP, brain natriuretic peptide; BUN, blood urea nitrogen; CRP, C‐reactive protein; DBP, diastolic blood pressure; FBG, fasting blood glucose; GH, glycosylated hemoglobin; GPT, glutamic‐pyruvic transaminase; HDL‐C, high‐density lipoprotein cholesterol; IRI, insulin resistance index; LDL‐C, low‐density lipoprotein cholesterol; SBP, systolic blood pressure; T2DM, type 2 diabetes mellitus.

* *P* < .05.

### 2.2 Data collection and biochemical measurements

After enrollment, a serial medical examination was carried out, including age, history of T2DM, blood pressure (diastolic [DBP] and systolic [SBP]), height, weight, waist circumference, and hip circumference. Body mass index (BMI) was calculated by weight/height^2^ (kg/m^2^). We collected venous blood from all study subjects after 10 hours' fasting. A fully automatic biochemical analyzer (AU‐640) was utilized to examine the liver function markers, total cholesterol, triglyceride, low‐density lipoprotein cholesterol (LDL‐C), high‐density lipoprotein cholesterol (HDL‐C), albumin, glycosylated hemoglobin, C‐reactive protein (CRP), renal function markers, and fasting glucose. Electro‐chemiluminescence assays were used to detect the fasting insulin and C peptide levels. Meanwhile, the plasma was collected from 3‐mL blood samples (centrifuged for 5 minutes at a speed of 1917 *g*) and stored at −80° for further analysis. Enzyme‐linked immunosorbent assay was used to examine adiponectin (No. ABIN364986, ACRIS) and chemerin (SEA945Hu, Cloud‐Clone) levels in plasma. Samples were first incubated in microtitration wells for 60 minutes, then washed and incubated with biotin‐labeled polyclonal anti‐human chemerin or adiponectin antibody at 37°C for 90 minutes. After another washing, streptavidin‐horseradish peroxidase conjugate was added and samples were incubated at 37°C for 30 minutes. The reaction was stopped with acidic solution (H2SO4). An enzyme standard instrument was used to detect the optical density (OD) value at 450 nm. Finally, we plotted a standard curve to calculate the chemerin and adiponectin concentrations of each sample. Meanwhile, the ba‐PWV, bone mineral density *T* value (DPX‐Bravo), and ankle‐brachial index (ABI) were examined as in previous studies.^10^, ^12^

### 2.3 Statistical analysis

All analyses were done with IBM SPSS Statistics Version 22.0. The data according to normal distribution are shown as mean ± SD, otherwise logarithmic transformation was carried out. Before further analysis, we first carried out an *F* test and a normality test. The Student's *t* test was utilized to assess the difference between different groups. Correlation analysis between two factors was analyzed with Pearson's test. *P* < .05 was considered as a statistically significant difference. Multiple linear stepwise regression analysis was applied to verify principal influence factors.

## 3 RESULTS

### 3.1 Evaluation of the correlation of ba‐PWV and several clinical parameters in T2DM patients

Several clinical parameters were collected and measured from all the enrolled patients as well as healthy controls (HCs). The insulin, insulin resistance index, and ba‐PWV levels were significantly higher in T2DM patients than in HCs (*P* < .05), which is consistent with our diagnosis in the inpatient clinic. The 120 T2DM subjects were separated into three groups according to the ba‐PWV level: the high‐, moderate‐, or low‐ba‐PWV groups. The clinical parameters were compared among the different groups. The high‐ba‐PWV group had elevated levels for SBP, BMI, glycosylated hemoglobin, CRP, brain natriuretic peptide (BNP), LDL‐C, and fasting glucose insulin, while its levels for albumin, HDL‐C, bone mineral density *T* value score, and ABI were significantly lower than those in the moderate‐ba‐PWV group (*P* < .05; Table 2). Compared with the low‐ba‐PWV group, the high‐ba‐PWV group showed higher levels for T2DM history, DBP, waist circumference, and insulin (*P* < .05; Table 2). Furthermore, lower levels for SBP, BMI, waist circumference, BNP, and LDL‐C were observed in the low‐ba‐PWV group, along with higher levels for HDL‐C, bone mineral density *T* value score, and ABI compared with the moderate‐ba‐PWV group (*P* < .05; Table 2). Interestingly, ABI, which protects generalized atherosclerosis at high levels, was negatively associated with ba‐PWV levels, and their combination has been suggested for better diagnosis of atherosclerosis.^13^ There were no differences in age, aspartate, aminotransferase, hip circumference, blood urea nitrogen, creatinine, uric acid triglycerides, total cholesterol, or C peptide among these groups (*P* < .05).

**Table 2:** Comparison of clinical parameters among low‐, moderate‐, and high‐ba‐PWV groups

Parameter	ba‐PWV (cm/s)			*P*
	High	Moderate	Low	
Case (Male/Female)	40 (23/17)	40 (22/18)	40 (25/15)	<.05
Age (y)	83.10 ± 6.57	84.88 ± 5.89	83.53 ± 8.89	>.05
History of T2DM (y)	22.95 ± 4.73^b^	21.68 ± 3.79	19.58 ± 5.62	<.05
SBP (mm Hg)	149.45 ± 9.30^a^, ^b^	139.75 ± 10.97^c^	132.03 ± 12.70	<.05
DBP (mm Hg)	79.65 ± 9.57^b^	76.58 ± 9.89	73.78 ± 7.10	<.05
BMI (kg/m^2^)	25.63 ± 3.40^a^, ^b^	24.04 ± 2.65^c^	22.23 ± 2.28	<.05
Waist circumference (cm)	99.58 ± 7.82^b^	97.65 ± 8.00^c^	94.13 ± 7.62	<.05
Hip circumference (cm)	99.25 ± 9.19	100.75 ± 8.32	102.03 ± 8.29	>.05
Waist‐to‐hip ratio	1.01 ± 0.05^a^, ^b^	0.97 ± 0.05^c^	0.92 ± 0.06	<.05
GPT (U/L)	20.83 ± 8.64	19.53 ± 7.98	21.10 ± 8.66	>.05
AST (U/L)	24.38 ± 7.73	22.03 ± 6.97	22.65 ± 7.94	>.05
BUN (mmol/L)	6.06 ± 2.13	6.54 ± 3.31	6.11 ± 1.45	>.05
Serum creatinine (µmol/L)	84.90 ± 26.93	80.03 ± 16.70	90.40 ± 30.40	>.05
Trioxypurine (µmol/L)	309.38 ± 51.87	310.05 ± 60.33	311.40 ± 61.64	>.05
GH (%)	6.56 ± 0.85^a^, ^b^	5.78 ± 1.36	5.75 ± 1.40	<.05
CRP (mg/L)	4.53 ± 2.53^a^, ^b^	2.29 ± 1.56	2.19 ± 3.40	<.05
Albumin (g/L)	37.26 ± 3.45^b^	38.39 ± 3.26	38.93 ± 3.56	<.05
BNP (pg/mL)	1533.80 ± 878.71^a^, ^b^	1023.03 ± 653.60^c^	543.80 ± 476.45	<.05
LDL‐C (mmol/L)	2.77 ± 1.17^a^, ^b^	2.22 ± 0.73^c^	1.84 ± 0.66	<.05
HDL‐C (mmol/L)	0.88 ± 0.24^a^, ^b^	1.12 ± 0.20^c^	1.32 ± 0.29	<.05
Triglyceride (mmol/L)	1.54 ± 0.65	1.40 ± 0.53	1.35 ± 0.53	>.05
Total cholesterol (mmol/L)	4.06 ± 1.19^b^	3.65 ± 0.73	3.46 ± 0.78	>.05
BMD *T* value	−2.88 ± 1.08^a^, ^b^	−1.62 ± 0.89^c^	−0.17 ± 1.19	<.05
ABI	0.82 ± 0.08^a^, ^b^	0.93 ± 0.10^c^	1.27 ± 0.64	<.05
C peptide (ng/mL)	2.09 ± 1.13	1.72 ± 0.94	1.94 ± 0.98	>.05
Insulin (pmol/L)	310.32 ± 197.61^a^, ^b^	125.13 ± 87.45	82.36 ± 110.76	<.05
FBG (mmol/L)	7.04 ± 1.69^a^, ^b^	5.66 ± 1.09	6.03 ± 2.53	<.05
IRI	15.02 ± 11.59^a^, ^b^	4.48 ± 3.15	4.01 ± 9.34	<.05

Abbreviations: ABI, ankle‐brachial index; AST, aspartate aminotransferase; ba‐PWV, brachial‐ankle pulse wave velocity; BMD *T* value, bone mineral density *T* value; BMI, body mass index; BNP, brain natriuretic peptide; BUN, blood urea nitrogen; CRP, C‐reactive protein; DBP, diastolic blood pressure; FBG, fasting blood glucose; GH, glycosylated hemoglobin; GPT, glutamic‐pyruvic transaminase; HDL‐C, high‐density lipoprotein cholesterol; IRI, insulin resistance index; LDL‐C, low‐density lipoprotein cholesterol; SBP, systolic blood pressure; T2DM, type 2 diabetes mellitus.

a High vs moderate, *P* < .05.

b High vs low, *P* < .05.

c Moderate vs low, *P* < .05.

### 3.2 Evaluation of the links between chemerin and adiponectin levels and ba‐PWV in T2DM patients

**Figure 1:**
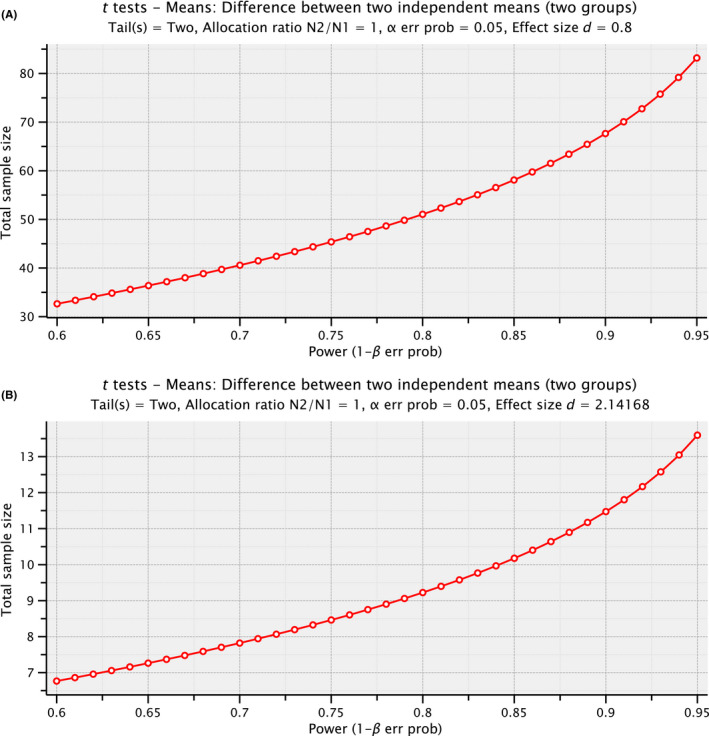
Power analysis of the study design using G*power analysis. A, Power analysis based on Cohen's *d* by using 0.8 as large effect size. The total sample size should reach ~50 to get the power at 0.8. B, Power analysis based on the data in the study and the default setting of G*power software to get an effect size at 2.14168, which needs a total sample size of at least 10 to reach the power at 0.8

First, power analysis was carried out according to Cohen's *d* by using G*Power software,^14^ and support our experiment design with the sample size analyzed in each group in this study (Figure 1). We compared the chemerin and adiponectin levels between HC older adults (n = 9) and T2DM patients (n = 120). T2DM patients had higher levels of chemerin than HC older adults (T2DM vs HC, 136.39 ± 20.98 vs 56.99 ± 10.07 ng/mL, *P* < .05) and lower levels of adiponectin were detected in T2DM patients (T2DM vs HC, 12.75 ± 4.56 vs 20.64 ± 2.52 µg/mL, *P* < .05). 

**Figure 2:**
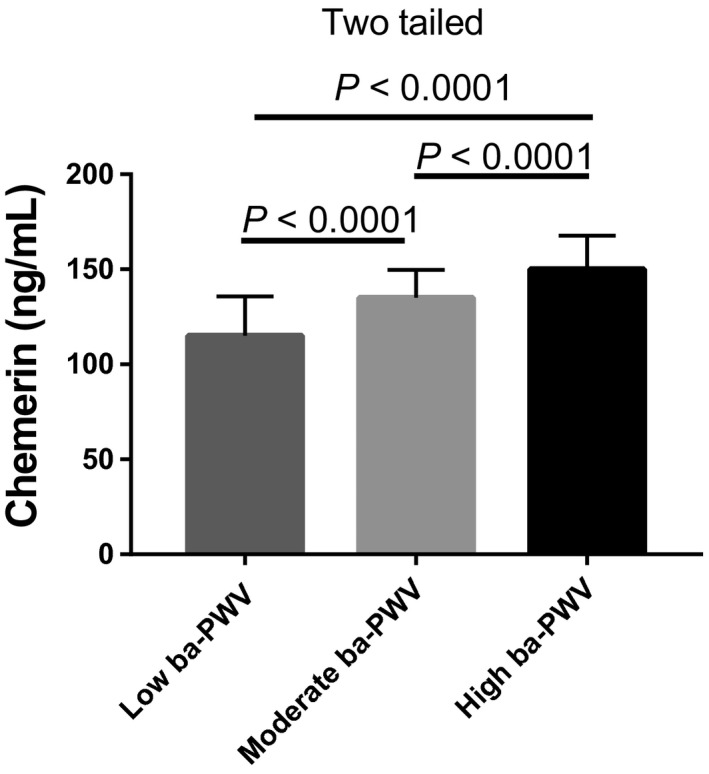
Comparison of chemerin levels among the low, moderate, and high brachial‐ankle pulse wave velocity (ba‐PWV) groups in type 2 diabetes mellitus patients. Significant differences were calculated with the Student's *t* test

**Figure 3:**
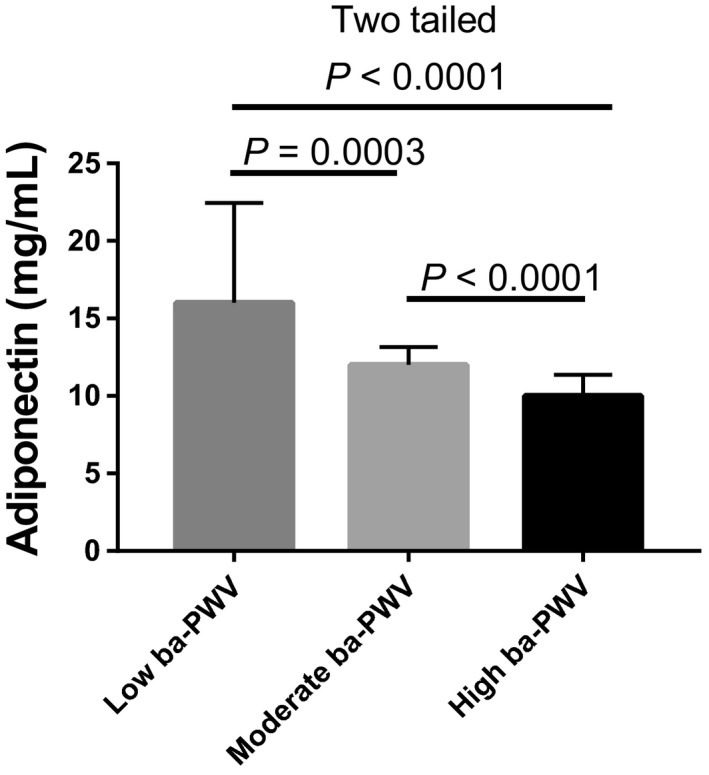
Comparison of adiponectin levels among the low, moderate, and high brachial‐ankle pulse wave velocity (ba‐PWV) groups in type 2 diabetes mellitus patients. Significant differences were calculated with the Student's *t* test

Next, the chemerin and adiponectin levels among these three groups were compared. Both the high‐ and moderate‐ba‐PWV groups showed higher levels of chemerin than the low‐ba‐PWV group (Figure 2). Interestingly, inverted results were observed for adiponectin production in the low‐ba‐PWV group (Figure 3). Furthermore, the correlation analysis showed that chemerin and adiponectin levels were significantly related to ba‐PWV levels (chemerin, *r* = 0.806, *P* < .001; adiponectin, *r* = −.554, *P* < .001; Table 3).

**Table 3:** Correlation between plasma chemerin or adiponectin and ba‐PWV levels

Parameter	ba‐PWV	
	Correlation (*r*)	*P* value
Adiponectin	−.554	<.001
Chemerin	.806	<.001

Abbreviation: ba‐PWV, brachial‐ankle pulse wave velocity.

### 3.3 Identification of the early diagnostic markers for vascular stiffness in T2DM patients

To investigate the potential markers for the prediction of atherosclerosis, we analyzed the correlation between chemerin levels and these measured clinical parameters in T2DM patients. As shown in Tables 4 and 5, chemerin and adiponectin levels in plasma were significantly related to history of T2DM, SBP, DBP, BMI, waist circumference, waist‐to‐hip ratio, glycosylated hemoglobin, CRP, BNP, LDL‐C, triglycerides, total cholesterol, insulin, fasting glucose, insulin resistance index, hip circumference, albumin, HDL‐C, bone mineral density *T* value score, and ABI (*P* < .001). Furthermore, our multiple linear regression analysis showed that chemerin and adiponectin levels were significantly associated with ba‐PWV in T2DM patients (Table 6, *P* < .001).

**Table 4:** Correlation between plasma chemerin and clinical parameters

Parameter	Chemerin		Parameter	Chemerin	
	Correlation (*r*)	*P* value		Correlation (*r*)	*P* value
History (y)	.164	.037	LDL‐C	.474	<.001
SBP (mm Hg)	.604	<.001	HDL‐C	−.570	<.001
DBP (mm Hg)	.262	.004	Triglyceride (mmol/L)	.152	.049
BMI (kg/m^2^)	.541	<.001	Total cholesterol (mmol/L)	.325	<.001
Waist circumference (cm)	.290	.001	BMD *T* value	−.763	<.001
Hip circumference (cm)	−.183	.023	ABI	−.475	<.001
Waist‐to‐hip ratio	.593	<.001	Insulin (µmol/L)	.510	<.001
GPT (U/L)	.416	<.001	FBG (mmol/L)	.324	<.001
CRP (mg/L)	.343	<.001	IRI	.473	<.001
Albumin (g/L)	−.261	.002	Adiponectin	−.527	<.001
BNP (pg/mL)	.474	<.001	ba‐PWV	.806	<.001

Abbreviations: ABI, ankle‐brachial index; ba‐PWV, brachial‐ankle pulse wave velocity; BMD *T* value, bone mineral density *T* value; BMI, body mass index; BNP, brain natriuretic peptide; CRP, C‐reactive protein; DBP, diastolic blood pressure; FBG, fasting blood glucose; GPT, glutamic‐pyruvic transaminase; HDL‐C, high‐density lipoprotein cholesterol; IRI, insulin resistance index; LDL‐C, low‐density lipoprotein cholesterol; SBP, systolic blood pressure.

**Table 5:** Correlation between plasma adiponectin level and clinical parameters

Parameter	Adiponectin		Parameter	Adiponectin	
	Correlation (*r*)	*P* value		Correlation (*r*)	*P* value
SBP (mm Hg)	−.351	<.001	HDL‐C	.420	<.001
DBP (mm Hg)	−.232	.011	BMD *T* value	.511	.020
BMI (kg/m^2^)	−.291	<.001	ABI	.673	<.001
Waist circumference (cm)	−.208	.010	Insulin (µmol/L)	−.284	<.001
Hip circumference (cm)	−.346	<.001	FBG (mmol/L)	−.177	.025
Waist‐to‐hip ratio	−.170	.032	IRI	−.250	.003
CRP	−.231	.005	Chemerin	−.527	<.001
BNP	−.333	<.001	ba‐PWV	−.554	<.001
LDL‐C	−.209	.010			

Abbreviations: ABI, ankle‐brachial index; BMD *T* value, bone mineral density *T* value; BMI, body mass index; BNP, brain natriuretic peptide; CRP, C‐reactive protein; DBP, diastolic blood pressure; FBG, fasting blood glucose; HDL‐C, high‐density lipoprotein cholesterol; IRI, insulin resistance index; LDL‐C, low‐density lipoprotein cholesterol; SBP, systolic blood pressure.

**Table 6:** Multiple regression analysis of chemerin with ba‐PWV levels

Model	Unstandardized coefficient		Standardized coefficient	*t*	*P*
	partial regression coefficient (*B*)	SE	β		
Constant	56.948	5.490		9.328	.005
ba‐PWV	0.022	0.003	0.545	6.445	<.001
BMD *T* value	−4.484	1.158	−0.327	3.553	.001

Abbreviations: ba‐PWV, brachial‐ankle pulse wave velocity; BMD *T* value, bone mineral density *T* value.

## 4 DISCUSSION

Abnormal adipose tissue secretes chemerin and adiponectin—pro‐inflammatory and anti‐inflammatory substances, which may link obesity to diabetes, atherosclerosis, and cardiovascular disease.^4^, ^15^, ^16^ In the present study, we observed higher levels of chemerin and lower levels of adiponectin in the high‐ba‐PWV T2DM group than in the low‐ and‐moderate‐ba‐PWV T2DM groups. Chemerin and adiponectin levels were significantly correlated with ba‐PWV levels. Furthermore, the presence of chemerin and adiponectin was significantly associated with the levels of several clinical parameters. Overall, this study suggests that plasma levels of chemerin and adiponectin are closely related to the development of vascular stiffness, and may be useful for the diagnosis of early stage atherosclerosis.

In this study, all enrolled T2DM patients showed different degrees of atherosclerosis, which provides more evidence supporting the theory that diabetes promotes the development of atherosclerosis.^17^ Similar results have been observed in previous reports: The ba‐PWV level is significantly correlated with several clinical parameters that are risk factors for atherosclerosis, such as glycosylated hemoglobin and CRP.^10^ PWV values represent a gold standard for measuring arterial stiffness, which is determined principally by age and blood pressure.^18^ Arterial stiffening and atherosclerosis share some common pathophysiological mechanisms, such as endothelial dysfunction and insulin resistance.^19^ In addition, insulin resistance has been connected to the constant arterial stiffening found in diabetic patients,^20^, ^21^ while the stiffness may be further increased by endothelial dysfunction and high levels of gamma‐glutamyltransferase and leptin, and by decrease in adiponectin levels involving mechanisms implicated also in preclinical atherosclerosis.^22^, ^23^, ^24^, ^25^ Our studies in T2DM patients showed similar findings to support this theory. Nevertheless, Maedeker et al^26^ suggested that the increased vascular stiffness may simply be a consequence of the pathological changes during the progression of atherosclerosis, which positively reinforces the link between stiffness and atherosclerosis.

Recently, many studies have pointed out that adipose tissue is an endocrine organ, rather than just functioning as a fuel repository.^27^, ^28^ Adipose tissue secretes a series of adipokines, such as leptin, tumor necrosis factor, interleukin‐6, angiotensin, chemerin, insulin‐like growth factor, and adiponectin, which diversifies their functions in obesity patients.^4^, ^28^ Our results showed that chemerin and adiponectin levels were significantly related to a history of T2DM and to ba‐PWV levels, suggesting that chemerin and adiponectin may play critical roles in the progression of T2DM and of vascular stiffness. Chemerin is one of the G‐protein‐coupled receptor (chemerin R23) ligands, which promote the function of mature fat cells, synthesize triglycerides, transport glucose, and inhibit lipid degradation, thus reducing blood sugar levels under normal circumstances.^29^, ^30^ Chemerin can promote cholesterol intake and foam‐cell formation, which causes atherosclerotic plaque formation.^31^, ^32^ Furthermore, chemerin also enhances HDL‐associated paraoxonase‐1 activity and triggers high‐grade inflammation and oxidative stress, which consequently increases the oxidized LDL level and induces atherosclerosis.^4^ Consistent with other findings, our results suggest that chemerin could be considered as a marker for subclinical vascular stiffness detection in T2DM patients.^10^ Adiponectin, an adipocyte‐specific secretory protein, is mainly regulated in endothelial inflammatory responses.^33^ In contrast to chemerin, adiponectin is important in transporting glucose, regulating lipid metabolism, and improving insulin sensitivity, which elicit an anti‐inflammatory role in inhibiting the formation of atherosclerosis.^4^, ^33^, ^34^ A previous study on rhesus monkeys with type 2 diabetes showed that adiponectin levels are decreased in parallel with reduced insulin sensitivity.^35^ Our results are similar to previous findings that adiponectin level in plasma is significantly related to subclinical vascular stiffness in asymptomatic patients with type 1 diabetes mellitus.^11^ In addition, the plasma levels of chemerin and adiponectin have been considered as risk factors of atherosclerosis and cardiovascular diseases.^10^, ^11^, ^33^, ^36^ Furthermore, ABI and ba‐PWV levels are crucial risk factors for cardiovascular diseases in patients with T2DM,^13^, ^37^ which should be considered for better prediction of atherosclerosis in T2DM patients. Thus, our data provide more support for utilizing chemerin and adiponectin levels as potential markers for vascular stiffness, together with ABI levels to better predict the early stage of atherosclerosis, and even for cardiovascular diseases.

In summary, chemerin and adiponectin levels in plasma are suggested as independent predictors for vascular stiffness, which promotes the development of subclinical atherosclerosis, and may be applied for the risk assessment of macrovascular diseases in clinical settings.

## CONFLICTS OF INTEREST

Nothing to disclose.
